# Biomarker development for neonicotinoid exposure in soil under interaction with the synergist piperonyl butoxide in *Folsomia candida*

**DOI:** 10.1007/s11356-022-21362-z

**Published:** 2022-06-21

**Authors:** Ruben Bakker, Astrid Ekelmans, Liyan Xie, Riet Vooijs, Dick Roelofs, Jacintha Ellers, Katja M. Hoedjes, Cornelis A. M. van Gestel

**Affiliations:** 1 Amsterdam Institute for Life and Environment (A-LIFE), Faculty of Science, Vrije Universiteit Amsterdam, De Boelelaan 1085, 1081 HV Amsterdam, The Netherlands; 2 Keygene N.V., Agro Business Park 90, Wageningen, 6708 PW The Netherlands

**Keywords:** Springtails, Imidacloprid, Thiacloprid, Quantitative real-time PCR, Piperonyl butoxide

## Abstract

Pesticide toxicity is typically assessed by exposing model organisms to individual compounds and measuring effects on survival and reproduction. These tests are time-consuming, labor-intensive, and do not accurately capture the effect of pesticide mixtures. Moreover, it is unfeasible to screen the nearly infinite combinations of mixtures for synergistic effects on model organisms. Therefore, reliable molecular indicators of pesticide exposure have to be identified, i.e., biomarkers. These biomarkers can form the basis of rapid and economical screening procedures to assess the toxicity of pesticides even under synergistic interaction with other pollutants. In this study, we screened the expression patterns of eight genes for suitability as a biomarker for neonicotinoid exposure in the soil ecotoxicological model *Folsomia candida* (springtails). Springtails were exposed to the neonicotinoids imidacloprid and thiacloprid either alone or with various levels of piperonyl butoxide (PBO), which inhibits cytochrome P450 enzymes (CYPs): a common point of synergistic interaction between neonicotinoid and other pesticides. First, we confirmed PBO as a potency enhancer for neonicotinoid toxicity to springtail fecundity, and then used it as a tool to confirm biomarker robustness. We identified two genes that are reliably indicative for neonicotinoid exposure even under metabolic inhibition of CYPs by PBO, *nicotinic acetylcholine receptor–subunit alpha 1* (*nAchR*) and *sodium-coupled monocarboxylate transporter* (*SMCT*). These results can form the basis for developing high-throughput screening procedures for neonicotinoid exposure in varying mixture compositions.

**Supplementary Information:**

The online version contains supplementary material available at 10.1007/s11356-022-21362-z.

## Introduction

Neonicotinoids are the most commonly used insecticides globally of the past three decades (Borsuah et al., 2020), but are harmful to non-target organisms like pollinators (Goulson, 2013; Pisa et al., 2014) and soil invertebrates (de Lima e Silva et al., 2017, 2020, 2021). As a consequence, ecosystem services crucial for sustainable agriculture, such as nutrient cycling, pest control, and pollination, are under threat by the use of neonicotinoid insecticides (EASAC, 2015; FAO, 2020; Gunstone et al., 2021).

Current environmental risk assessment (ERA) and policy regarding pesticides is based on phenotypic toxicity tests that measure effects on the survival and reproduction of model organisms after exposure to individual pesticides. Extrapolation of these findings to ecotoxicological effects in the field is difficult as most agricultural soils are polluted by pesticide mixtures (Pelosi et al., 2021; Silva et al., 2019), and the synergistic interactions between pesticides within mixtures is a major knowledge gap (Gunstone et al., 2021). Furthermore, the predicted effect concentrations derived from these phenotypic tests can only be used in ERA after measuring the exposure concentration of the pollutants in soil, a laborious and costly procedure. On the contrary, gene expression responses can be used to determine the type of pollution even under varying mixture composition (Fontanetti et al., 2011; Shi et al., 2017). Determining the effects of the near infinite number of possible soil pollution mixtures on the gene expression of model organisms is unfeasible. Therefore, reliable genetic responses, i.e. biomarkers, have to be identified that remain indicative for a group of soil pollutants even under synergistic interaction with other pollutants. Gene expression biomarkers, in turn, can be used in biomonitoring; a cost-effective tool to screen for samples that, in case of detecting a potential risk, may be subjected to subsequent chemical analysis to identify the chemical(s) of concern. In this way, gene expression assays may provide ERA with more accurate metrics of adverse effects by pesticides than traditional toxicity tests.

The selection of candidate gene expression patterns requires an understanding of the molecular mediators behind pesticide toxicity in a relevant non-target model organism. Most studies on the molecular mechanisms that mediate neonicotinoid toxicity in invertebrates have been carried out in honey bees. However, the honey bee is not an ideal representative for non-target soil invertebrates because it does not live in the soil, its genome is limited in its detoxification capacity (Claudianos et al., 2006), and it has an unusual life history due to its social lifestyle (Gradish et al., 2019). *Folsomia candida* is a more suitable representative for non-target soil invertebrates because (1) it belongs to the springtails (Collembola), which is one of the most prevalent non-target invertebrate groups (Rusek, 1998), and a key component of the soil food web by promoting nutrient cycling (FAO, ITPS, GSBI, 2020); (2) *F. candida* is well established as a soil ecotoxicological model species since the 1960s (van Gestel, 2012); (3) its genome has been sequenced and annotated facilitating the development of molecular tools for studying its genomic responses to pollution (Faddeeva-Vakhrusheva et al., 2017), and (4) *F. candida* is representative for the sensitivity to neonicotinoids of other springtail species (de Lima e Silva et al., 2021). Together, these aspects make *F. candida* an ideal candidate for the development of biomarker assays for the monitoring of pesticide exposure in soil.

For the successful applications of neonicotinoid biomonitoring, gene expression patterns have to be identified that are indicative for the exposure to a variety of neonicotinoids and remain to do so even under synergistic interaction with other pollutants. Neonicotinoids are commonly subdivided in two groups, depending on the inclusion of either nitro- or cyano-moieties into their chemical structure (Buszewski et al., 2019). Although both groups share the same mode-of-action, the nitro-substituted neonicotinoids are more toxic than the cyano-substituted ones to the fecundity and survival of various springtail species (de Lima e Silva et al., 2017, 2020; 2021). In the honey bee, the differential toxicity of the two groups of neonicotinoids has been attributed to an increased detoxification rate of the cyano-substituted ones by CYP enzymes (Iwasa et al., 2004; Manjon et al., 2018). Moreover, CYP inhibition has also been proposed to trigger synergistic interactions between neonicotinoids and other pesticides such as triazole fungicides (Feyereisen, 2018; Glavan & Bozic, 2013; Raimets et al., 2017; Sgolastra et al., 2017). Finally, various studies on the genomic response of *F. candida* to various pollutants have identified CYP genes as biomarkers for a variety of chemicals (Chen et al., 2014; de Boer et al., 2009; Nota et al., 2009; Qiao et al., 2015; Roelofs et al., 2012). Based on these findings, CYPs have emerged as promising biomarkers for the toxicity of neonicotinoid exposure. Yet, it remains to be confirmed if expression patterns of CYP genes provide a reliable indication for the toxicity of both cyano- and nitro-substituted neonicotinoids, as well as for synergistic interaction with other pesticides. This also needs to be confirmed still for other biomarkers identified for neonicotinoid exposure in the honey bee (Christen et al., 2016; Fent et al., 2020; Manjon et al., 2018). Given the central role of CYPs in mediating differential effects of the two major classes of the neonicotinoid family and their role in mediating synergy, we propose inhibition of CYPs could serve as “stress-test” to assess biomarker robustness. For this, we applied piperonyl butoxide (PBO), which is a CYP inhibitor that forms a metabolite-inhibitory complex with CYPs and thereby prevents the binding of other substrates (Hodgson & Levi, 1999). By choosing PBO over toxicants, we can ensure that observed effects on biomarker gene expression are the result CYP inhibition, rather than, other synergistic interactions.

The integration of multiple biomarkers into a panel for biomonitoring and ERA is highly recommended, because the range of effects soil pollution has on organisms is diverse (Lionetto et al., 2019). The aim of this study was to assess the suitability of candidate genes to construct a panel of biomarkers for the assessment of soil polluted with neonicotinoids. For this, we considered three criteria: (1) the panel should indicate exposure of both nitro- and cyano-substituted neonicotinoids, (2) the response of the panel should relate in a concentration-dependent manner with the adverse fitness effect of neonicotinoid exposure on *F. candida*, and (3) the expression patterns of biomarkers in the panel should be reliable under synergistic interaction caused by CYP inhibition by PBO. To represent the two major classes of neonicotinoids, we selected imidacloprid and thiacloprid, as representatives of nitro- and cyano-substituted neonicotinoids, respectively. First, we determined the effect of PBO on the fecundity of springtails and its potency-enhancing effects when combined with thiacloprid and imidacloprid. Then, we screened the expression of eight candidate biomarker genes at various PBO and neonicotinoid concentrations using RT-qPCR. These were derived from previous studies on the genomic response of *F. candida* to various pollution types, which have identified gene expression patterns that may have potential to be applied as biomarkers (de Boer et al., 2009; Nota et al., 2009; Qiao et al., 2015; Roelofs et al., 2012).

## Materials and methods

### Test animals

*Folsomia candida* culture has been maintained by the A-LIFE section Ecology & Evolution of the Vrije Universiteit Amsterdam for > 20 years. The culture is kept in the dark at 16 ± 1°C and 75% relative air humidity (RH). The culture was reared in 1000 ml polypropylene containers with approximately 2 cm deep substrate of moistened activated charcoal and Paris plaster, at a 1:8 ratio, and continuously fed *ad libitum* with instant baker’s yeast (Algist Bruggeman N.V., Ghent, Belgium). To obtain age-synchronized individuals, batches of approximately 30 adults were sampled from the culture and placed in 125-ml translucent polypropylene containers filled with a 2-cm deep layer of the aforementioned substrate and covered with perforated lids to allow air flow. These were kept at 20 ± 1°C, 75% RH, and a 16:8 light-to-dark regime for about 48 h to allow egg-laying. After this period, the adults were removed and the substrate frequently moistened with demineralized water up to the point of saturation until the eggs hatched, about 10 days after egg-laying. The age-synchronized juveniles were fed with baker’s yeast and the substrate was moistened three times a week.

### Chemicals and test soil

Thiacloprid and imidacloprid, both 98% pure, were provided by Bayer CropScience, Monheim, Germany. Piperonyl butoxide (PBO; 90% pure) was obtained from Sigma-Aldrich, the Netherlands. All tests were carried out in natural LUFA 2.2 soil, Lufa Speyer, Germany. Soil attributes as determined by the supplier were total organic carbon content 2.1%, water-holding-capacity (WHC) 46.5% (w/w), and soil pH 5.5 (0.01 M CaCl_2_).

To spike the soil with thiacloprid or imidacloprid, stock solutions in demineralized water were thoroughly mixed in with dry soil to reach a moisture content of 22% of its dry weight, corresponding with 50% of its WHC. Thiacloprid was first dissolved in acetone amounting to approximately 3% of the stock solution volume before adding ultra-pure water. Imidacloprid was directly dissolved in ultra-pure water. Before use, stock solutions of both test chemicals were left overnight and stirred at 300 rounds per minute, at room temperature and covered with aluminum foil.

For PBO treatments, 15 g or 10% of the dry soil per treatment was placed into 100-ml glass jars wrapped with aluminum foil. The soil was submerged in a PBO-acetone solution and stirred every half hour for 2 h, after which it was left overnight in the fume hood to allow complete evaporation of the acetone. Then, the remaining soil for a treatment was added, mixed, moistened to 50% of its WHC, and again mixed thoroughly. In all tests, acetone controls were included as well as water controls that were not pretreated with acetone. All other treatments had 10% of their dry soil undergoing acetone pretreatment as described above.

Soils were prepared one day before the springtails were added. The concentration ranges used for single exposure to PBO were 0, 100, 200, 400, 600, 800, and 1000 mg kg^−1^ dry soil. For mutual exposure with neonicotinoids: PBO 0, 1, and 10 mg kg^−1^ dry soil was combined with thiacloprid at 0, 0.25, 0.5, 1, 2, 4, 8, and 16 mg kg^−1^ dry soil or imidacloprid at 0, 0.05, 0.1, 0.2, 0.4, 0.8, 1.6 mg kg^−1^ dry soil. For the gene expression assays, soil was spiked at 0, 10, and 100 mg PBO kg^−1^ dry soil (all concentrations << EC_1_), and combined with either 0, 0.1, 0.2, and 0.4 mg imidacloprid kg^−1^ dry soil or 0, 0.5, 1, and 2 mg thiacloprid kg^−1^ dry soil. The neonicotinoid concentrations for the gene expression assays were chosen to represent EC_1_, EC_10_, and EC_50_ values for reproduction effects of imidacloprid and thiacloprid from previous studies and fall within the proposed application concentrations of neonicotinoids (de Lima e Silva et al., 2017, 2020; 2021).

To determine the accuracy of soil spiking, 3–5 g of soil were sampled and stored at −20°C immediately after moistening and mixing and at the end of the toxicity tests. A selection of four samples taken before and one taken at the end of the toxicity test were analyzed by Groen Agro Control, Delfgauw, the Netherlands, following certified analytical methods. Detection limit was 0.01 mg kg^−1^ dry soil.

### Toxicity tests

Toxicity tests followed OECD guideline 232 for collembolan reproduction testing in soil (OECD, 2016) with the exception that the age of the animals was 21–23 days instead of 11–13 days after hatching and the test duration was reduced from 28 to 21 days.

Ten age-synchronized animals were added together with roughly the same number of grains of baker’s yeast to each 100-ml glass test jars containing approximately 30-g moist test soils. Every week, the water content of the soil was maintained using demineralized water and yeast was added when depleted. Toxicity tests were conducted at 20 ± 1°C, 75% RH, and a 16:8 light-dark regime. The tests were terminated by waterlogging the content of each jar and decanting it into 300-ml polypropylene beakers. Jars were rinsed to ensure all its content was collected in the beakers. The beakers were then stirred and left to rest for at least 5 min to allow all animals to float to the surface. Then, the surface was photographed by a Nikon Coolpix P510, and the adult and juvenile *F. candida* on the pictures were counted with Image J-based software Fiji (version Image.J 1.52p) using the Cell Counter plugin (Kurt de Vos, version from 2010).

### Gene expression analysis

Thirty age-synchronized springtails, i.e., 21–23 days after hatching, were exposed to soils spiked as described above. No food was added. After 48 h, the jars’ content was waterlogged. The springtails were scooped from the water surface into separate containers using a fine mesh sieve and transferred into 1.5-ml reaction tubes using an aspirator. The reaction tubes were snap frozen with liquid nitrogen and stored at −80°C. RNA was extracted with the SV Total RNA extraction kit (Promega, USA), following the manufacturer’s guidelines. Purity and quantity of total RNA was assessed by spectrophotometric measurements using a Nanodrop (Thermo-Fisher). The quality was checked on a 1% agarose gel containing 0.5% ethidium bromide. Approximately 500 ng of RNA was reverse transcribed into cDNA using Promega MML-V reverse transcriptase kit, following the manufacturer’s instructions. To verify DNA contamination, a no cDNA sample was prepared for one out of seven samples by omitting reverse transcriptase from the reactions. Quantitative PCR (qPCR) analysis was performed on a CFX Connect Real Time PCR Detection System (BIO-RAD, USA), using BIO-RAD 96 well plates and Cyber Green mix. The selected genes consisted of (1) three *Cytochrome P450 monooxygenases* (CYPs) that are affected by PBO enzymatic inhibition: *CYP3A13* and *CYP6e2*, which are involved in biotransformation of xenobiotics, and the CYP *methyl farnesoate epoxidase* (*FE*), which is involved in the maturation of juvenile hormone III; (2) markers for the action of neonicotinoids on neural signaling: *nicotinic acetylcholine receptor–subunit alpha1* (*nAchR*), which is the direct target of neonicotinoid activation, and *sodium-coupled monocarboxylate transporter 1* (*SMCT*) involved in the transmembrane transport of monocarboxylates such as nicotinate; and (3) adverse effect indicators: *heat shock protein 70* (*HSP70*), a general stress response protein; *isopenicillin N synthase* (*IPNS*), which catalyzes the formation of isopenicillin and response to stress; and a marker for fecundity: *vitellogenin-1* (*VIT*), which is required for egg yolk formation and transport. Primer sequences are listed in Table S1 with reference annotations according to Ensembl Metazoa version 50 (Cunningham et al., 2019). The primers of SMCT and nAchR were designed using the tool Primer Blast (Ye et al., 2012). The other primers were taken from previous studies (de Boer et al., 2009; Roelofs et al., 2012). All samples were run in comparison to two reference genes, i.e., *tyrosine 3-monooxygenase* (*YWHAZ*) and *eukaryotic transcription initiation factor 1A* (*ETIF*), and a no template and a no cDNA measurement. All measurements were performed in duplicate and measurements were rejected and repeated when they differed by half a threshold cycle (Ct). In case the measurements of either reference gene differed by half a threshold cycle (Ct), measurements for all primer sets were repeated for that sample.

### Data analysis

Data analysis was performed in *R* 4.0.0 (R Core Team, 2019). Graphics were generated via *ggplot2* (Wickham, 2016). Concentration-response curves were fitted using the R-package *drc* (Ritz et al., 2015), following the three-parameter logistic dose-response model. The EC_50_ values for the toxicity of imidacloprid and thiacloprid for the various levels of PBO exposure were compared using a likelihood ratio test.

The relative potencies, expressed as the ratio of ECx values at different PBO levels, were also calculated by the *drc* package in R as described in Ritz et al. (2006), with the 95% confidence intervals estimated using the delta method (Beckman & Weisberg, 1987) to determine deviation from 1.

General additive models (GAMs) were fitted over the log2-transformed gene expression values and analyzed using the R-package *mgcv* (Wood, 2011). Two models were fitted. The null model only took into consideration the influence of neonicotinoid exposure (equation 1), the full model did include the influence of neonicotinoid and PBO exposure (equation 2).

1 $$E={g}^{-1}\left({\beta}_0+\sum_{j=1}^{k_1}{\beta}_1{s}_j\left({X}_j\right)\right)$$

2 $$E={g}^{-1}\left({\beta}_0+\sum_{j=1}^{k_1}{\beta}_j{s}_j\left({x}_j\right)+\sum_{p=1}^{k_2}{\beta}_p{s}_p\left({X}_p\right)\right)$$

in which *E* is the expected value of the log2-normalized expression values, g^−1^ the inverse linkage function, *β*_0_ the intercept, *β*_*j*_ and *β*_*p*_ the coefficients for neonicotinoid (j) and PBO exposure (*p*), *s*_*j*_ and *s*_*p*_ smooth terms for neonicotinoid (*j*) and PBO exposure (*p*), and *k* the basis size.

Error was assumed normally distributed by selecting Gaussian family models and the smooth terms were estimated by restricted maximum likelihood (REML). The basis size (*k*) of the smooth terms (*s*) was set to maximum, i.e., to four for *s*_*j*_, the neonicotinoid smooth term (*k*1), and three for *s*_*p*_, the PBO smooth term (*k*2) (equations 1 and 2). Model fit was checked via numerous metrics. Residuals were inspected visually to see adherence to homogeneity using quantile-quantile plots and a histogram frequency plot of the residuals. The three models were compared using an *F*-test (Table S2). Full model was accepted when the *p*-value was lower than 0.1. The *p*-values per smooth term were determined at default by *mgcv* via *F*-tests.

## Results

### Soil concentrations

Chemical concentrations were measured in test soils spiked at concentrations around the EC_50_ for the toxicity of imidacloprid (0.4 mg kg^−1^ dry soil) and thiacloprid (1 mg kg^−1^ dry soil). The measured concentration of imidacloprid was on average 45% higher than the nominal one, and concentrations at the beginning and end of the exposure period were similar. The measured concentration of thiacloprid was 1.3% lower than the nominal one, and decreased to 31% of its original concentration at the end of the 21-day test period. Across both neonicotinoid exposures, PBO was detected at concentrations between 66 and 119% of the nominal ones. PBO degraded to about 57% of its original concentration at the end of the exposures (Table S3). All effect values are based on nominal concentrations.

### Effects of neonicotinoids and PBO on springtail fecundity

All controls, including the ones treated with acetone or with 1 and 10 mg PBO kg^−1^ dry soil, met the validity criteria set out by the OECD guideline 232, which are >80% adult survival, >100 juveniles, and a variation in juvenile numbers <30 % (Table S4). In the 1 mg kg^−1^ PBO reference group of the thiacloprid test, the coefficient of variance of juvenile numbers was slightly above the limit with 34% (Table S-4). To facilitate visual comparison of the concentration-response curves, all juvenile counts are shown as a percentage of the respective reference group mean.

PBO and the neonicotinoids did not cause sufficient mortality at the highest test concentrations to enable calculating LC_50_ values. PBO reduced the number of juveniles by 1% (EC_1_) at 288 mg kg^−1^ dry soil, and had an EC_10_ of 424 and an EC_50_ of 602 mg PBO kg^−1^ dry soil (Figure S1).

**Fig. 1:**
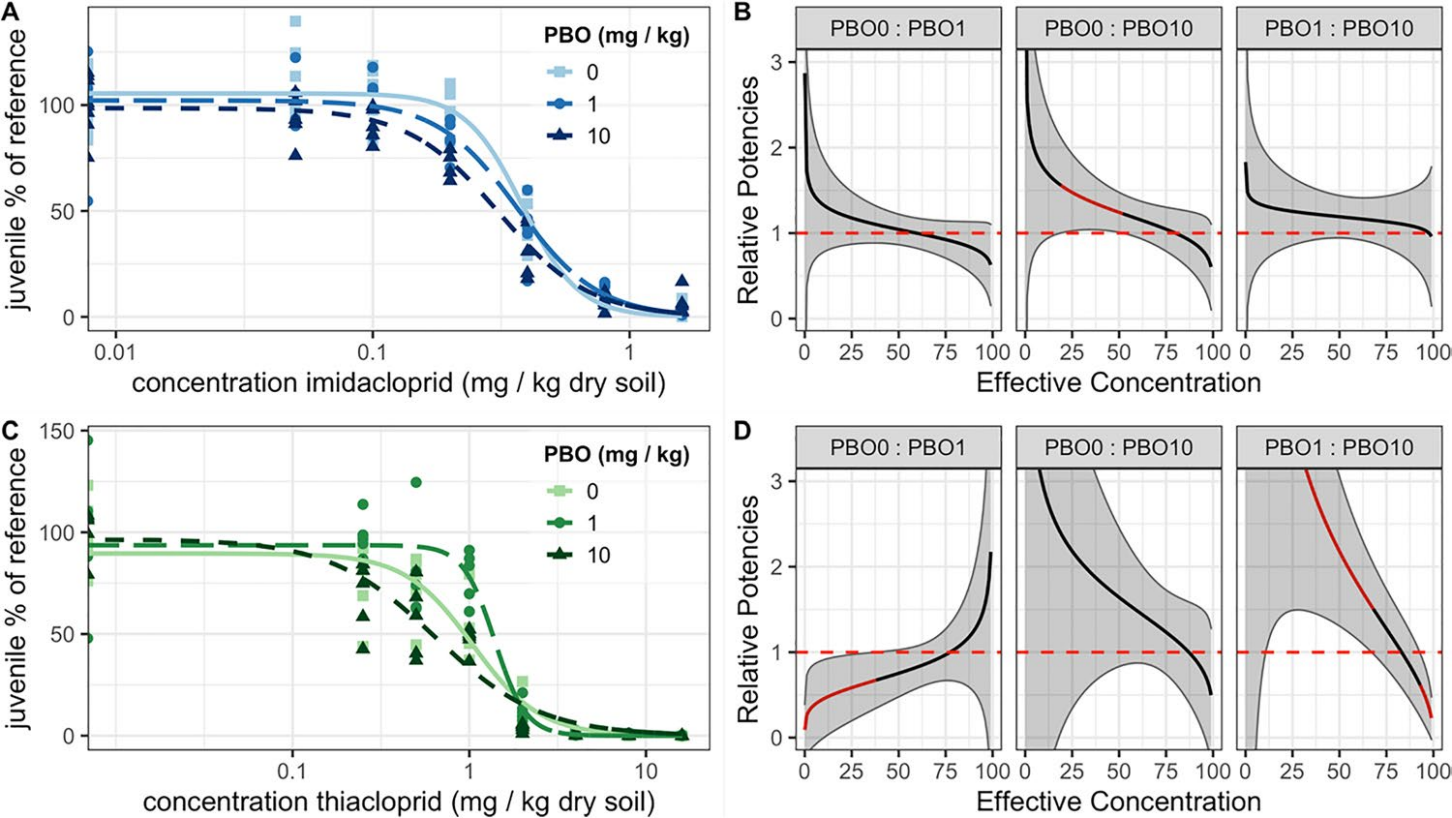
The effect of piperonylbutoxide (PBO) on the toxicity of the neonicotinoids imidacloprid (**A, B**) and thiacloprid (**C**, **D**) to the fecundity of the springtail Folsomia candida after 21 days exposure in LUFA 2.2 soil. Panels **A** and **C** show the fit to the data of the three-parameter concentration-response model for exposures to imidacloprid (panel **A**; blue) and thiacloprid (panel **C**; green) at various levels of PBO: solid lines and squares for 0 mg kg^−*1*^ dry soil, long-dashed lines and circles for 1 mg PBO kg^−*1*^ dry soil, and short-dashed lines and triangles for 10 mg PBO kg^−*1*^ dry soil. In panels **A** and **C**, the numbers of juveniles produced by the springtails are shown as a percentage of the reference group means. Panels **B** and **D** show the relative potencies of the neonicotinoids comparing the PBO regimes as indicated in the portrait headers. Solid black lines follow the relative potencies, and 95% confidence intervals calculated using the delta method are shown in gray bands outlined with gray lines. When the relative potencies deviated from equal potencies, i.e., the confidence interval not overlapping with 1 toxic unit, lines are shown in red indicating a significant effect of PBO addition on the toxicity of the neonicotinoid. The dashed red line indicates equal potency

EC_1_, EC_10_, and EC_50_ values for the effects on imidacloprid on juvenile numbers were 0.11, 0.21, and 0.37 mg kg^−1^ dry soil, respectively (Table 1). The concentration-response curves showed higher juvenile counts for the treatment of 0 mg PBO kg^−1^ dry soil, and intermediate effects for 1 mg PBO kg^−1^ dry soil. The lowest juvenile counts were observed for 10 mg PBO kg^−1^ dry soil, see Fig. 1A. The relative potency of imidacloprid at 10 mg PBO kg^−1^ dry soil was significantly increased compared to 0 mg PBO kg^−1^ dry soil between the 19 and 51% relative potencies: see Fig. 1B. The likelihood ratio test showed that PBO did not significantly affect the EC_50_ of imidacloprid (*df*_3,_ LR = 5.88, *p* = 0.12, loglikelihood ratio test).

**Table 1:** Toxicity of piperonyl butoxide (PBO) and its effect on the toxicity of imidacloprid and thiacloprid to Folsomia candida after 21 days exposure in LUFA 2.2 soil. EC_1_, EC_10_, and EC_50_ are effective concentrations reducing juvenile numbers by 1, 10, and 50% compared to the control, respectively. Values in parenthesis are 95% confidence intervals calculated using the delta method

**Exposure**	**PBO** **(mg kg**^**−1**^ **dry soil)**	**EC**_**1**_ **(mg kg**^**−1**^ **dry soil)**	**EC**_**10**_ **(mg kg**^**−1**^ **dry soil)**	**EC**_**50**_ **(mg kg**^**−1**^ **dry soil)**
PBO	*NA*	288 (160–418)	424 (324–524)	602 (544–660)
Thiacloprid	0	0.14 (−0.1 to 0.38)	0.40 (0.02–0.78)	1.0 (0.70–1.4)
	1	0.53 (0.26–0.81)	0.88 (0.62–1.1)	1.4 (1.2–1.6)
	10	0.03 (−0.01 to 0.06)	0.14 (0.03–0.25)	0.63 (0.40–0.87)
Imidacloprid	0	0.11 (0.05–0.17)	0.21 (0.15–0.27)	0.37 (0.33–0.41)
	1	0.06 (0.03–0.10)	0.16 (0.11–0.20)	0.36 (0.31–0.40)
	10	0.04 (0.01–0.07)	0.12 (0.07–0.16)	0.30 (0.25–0.35)

Thiacloprid affected springtail reproduction with EC_1_, EC_10_, and EC_50_ values of 0.14, 0.40, and 1.0 mg kg^−1^ dry soil, respectively (Table 1). The concentration-response curves (Fig. 1C), and EC_x_ values (Fig. 1D) show an increase in the potency of thiacloprid at 10 mg PBO kg^−1^ dry soil and a reduced potency at 1 mg PBO kg^−1^ dry soil. The effect of PBO on the EC_50_ was significant (*df*_3,_ LR = 19.34, *p* = 0.0002, loglikelihood ratio test). The influence of PBO on the potency of thiacloprid was in particular pronounced at low concentrations, i.e., between 0 and 0.5 mg thiacloprid kg^−1^ dry soil.

The direct comparison of the effect of PBO on the potency of imidacloprid and thiacloprid was hampered by the rather large variation in juvenile numbers in the reference groups of the thiacloprid tests. We assume it is coincidental and probably due to high variability in the control responses which is common in *F. candida* toxicity tests (Crouau & Cazes, 2003). Therefore, we compared models constrained and unconstrained in their EC_50_-values and calculated relative potencies between PBO exposure levels. This allows determining differential toxicity of compounds even when the control groups are dissimilar (Ritz et al., 2006, 2015).

### Effects of neonicotinoids and PBO on biomarker gene expression

**Fig. 2:**
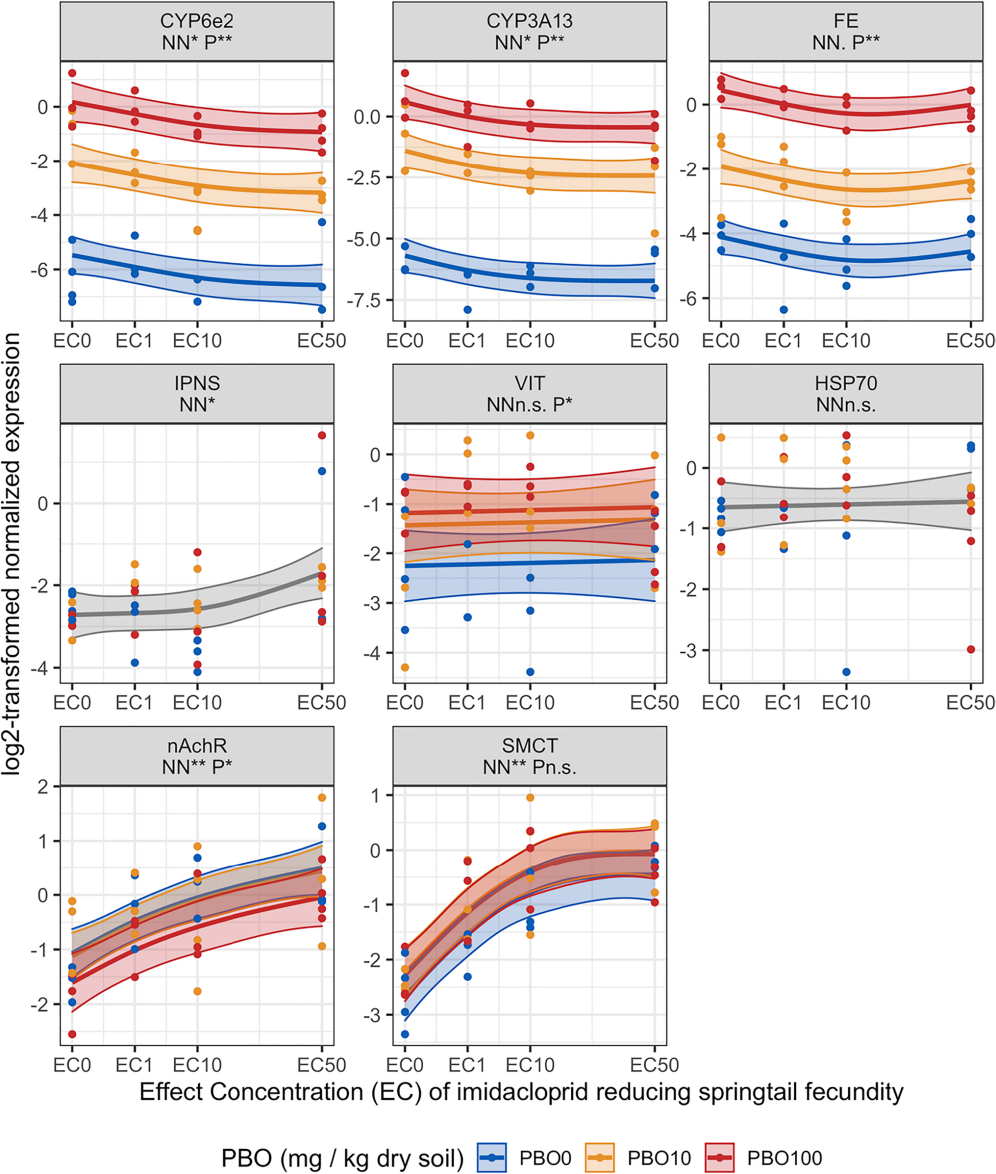
The influence of piperonylbutoxide (PBO) on the effect of imidacloprid on the gene expression of the springtail *Folsomia candida* exposed for 48 h in LUFA 2.2 soil. Imidacloprid concentrations are depicted as reference groups without imidacloprid, EC_0_, and the effect concentrations (EC) reducing the number of juveniles by 1, 10, and 50%, i.e., EC_1_, EC_10_, and EC_50_. Each panel represents the results of one gene, the names listed in the portrait headers are abbreviations for: cytochrome P450 monooxygenases (CYP) 3A13, CYP6e2, methyl farnesoate epoxidase (FE), heat shock protein 70 (HSP70), isopenicillin N synthase (IPNS), vitellogenin-1 (VIT), nicotinic acetylcholine receptor–subunit alpha1 (nAchR), and sodium-coupled monocarboxylate transporter 1 (SMCT). Below the names are the significance levels of the general additive model (GAM) smooth terms of neonicotinoid (NN) and PBO (P), depicted by the following symbols: p>0.1 “N.S”, p<= 0.1 “.”, p<= 0.05 “*”, p<=0.01 “**”. GAM mean functions are shown in solid lines, 95% confidence intervals as outlined transparent bands and dots depict the log2-transformed normalized expression values. PBO exposure levels are shown in blue, orange, and red for 0, 10, and 100 mg PBO kg^−*1*^ dry soil. Mean function and confidence interval outlined bands are shown in gray when the influence of PBO was not included in the GAM model fit

Imidacloprid suppressed the expression of all three CYPs (*CYP6e2*, *CYP3A13*, and *FE*), but did not exert significant effects on *HSP70* and *VIT* expression (Fig. 2). *IPNS* was upregulated by imidacloprid; although the pattern did not relate linearly with an increase of neonicotinoid exposure but rather reflected the variation within the data at the highest imidacloprid concentration (0.4 mg kg^−1^ dry soil), see Figure S2. Imidacloprid strongly upregulated the expression of *nAchR* and *SMCT* in a concentration-dependent manner (Fig. 2). For *SMCT*, we observed a concentration-dependent upregulation by imidacloprid until a concentration of 0.2 mg kg^−1^ dry soil, where after gene regulation remained at the same level.

**Fig. 3:**
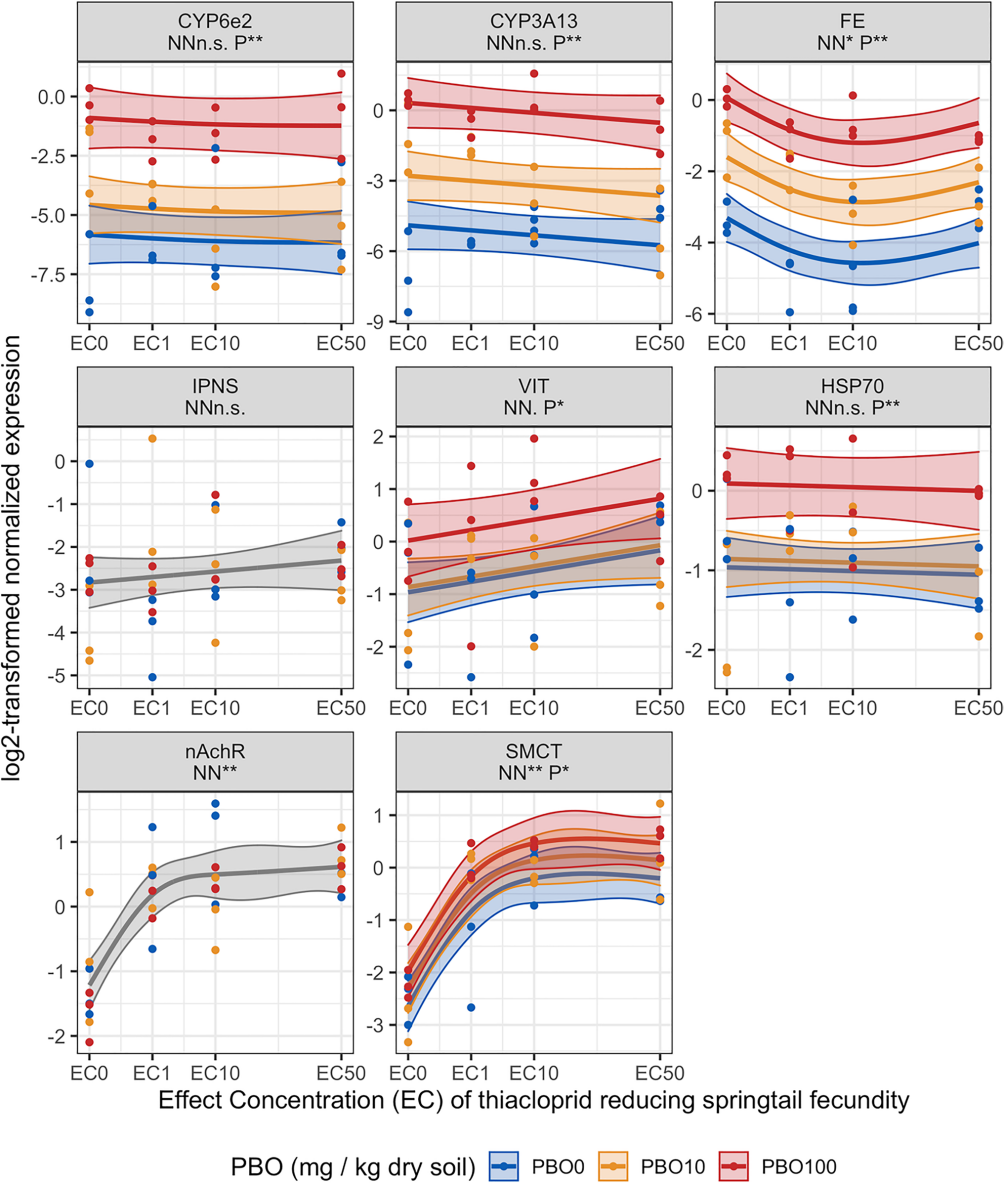
The influence of piperonylbutoxide (PBO) on the effect of thiacloprid on the gene expression of the springtail *Folsomia candida* exposed for 48 h in LUFA 2.2 soil. Thiacloprid concentrations are depicted as reference groups without thiacloprid, EC_0_, and the effect concentrations (EC) reducing the number of juveniles by 1, 10, and 50%, i.e., EC_1_, EC_10_, and EC_50_. Each panel represents the results of one gene, the names listed in the portrait headers are abbreviations for: *cytochrome P450 monooxygenases* (CYP) 3A13, CYP6e2, *methyl farnesoate epoxidase* (FE), *heat shock protein 70* (HSP70*), isopenicillin N synthase* (IPNS), *vitellogenin-1* (VIT), *nicotinic acetylcholine receptor–subunit alpha1* (nAchR), and *sodium-coupled monocarboxylate transporter 1* (SMCT). Below the names are the significance levels of the general additive model (GAM) smooth terms of neonicotinoid (NN) and PBO (P), depicted by the following symbols: *p*>0.1 “N.S”, *p*<= 0.1 “.”, *p*<= 0.05 “*”, *p*<=0.01 “**”. GAM mean functions are shown in solid lines, the 95% confidence intervals are shown as outlined transparent bands and dots depict the log2-transformed normalized expression values. PBO exposure levels are shown in blue, orange, and red for 0, 10, and 100 mg PBO kg^−1^ dry soil. Mean function and confidence interval outlined bands are shown in gray when the influence of PBO was not included in the GAM model fit

Thiacloprid did not influence the expression of *CYP6e2*, *CYP3A13*, *IPNS*, and *HSP70* (Fig. 3). *FE* expression was inhibited by thiacloprid exposure until 1 mg kg^−1^ soil and subsequently expression returned to control expression levels. *VIT* was upregulated by thiacloprid. Thiacloprid strongly upregulated the expression of *nAchR* and *SMCT* in a concentration-dependent manner, up to concentrations of 1 and 2 mg thiacloprid kg^−1^ dry soil after which gene expression levels remained at the same level.

PBO exposure strongly enhanced the expression of all CYPs when co-exposed with both neonicotinoids (Figs. 2 and 3). For all CYPs, the effect of PBO on gene expression was greater than the influence of the neonicotinoids, as determined by the significance levels of the GAM smooth term coefficients; Figs. 2 and 3. *VIT* was upregulated by PBO in a concentration-dependent manner under co-exposure of both neonicotinoids. *HSP70* and *SMCT* were upregulated by PBO under mutual exposure with thiacloprid (Fig. 3). For *HSP70*, upregulation occurred at the highest concentration of PBO (10 mg kg^−1^ dry soil). PBO did not influence *HSP70* and *SMCT* under mutual exposure with imidacloprid. *nAchR* was downregulated by PBO under mutual exposure with imidacloprid in particular at the highest concentration of PBO at 10 mg kg^−1^ dry soil (Fig. 1). *nAchR* was not affected by PBO exposure under mutual exposure with thiacloprid (Fig. 2).

## Discussion

Cytochrome P450 enzymes (CYP) are important mediators of differential toxicity between nitro- and cyano-substituted neonicotinoids in bees (Beadle et al., 2019; Iwasa et al., 2004; Manjon et al., 2018) and form a probable point of molecular synergistic interaction between neonicotinoids and triazole fungicides (Feyereisen, 2018; Glavan & Bozic, 2013; Raimets et al., 2017; Sgolastra et al., 2017). Therefore, we proposed the use of PBO as a “stress-test” to assess the reliability of biomarkers in indicating the exposure of the two major neonicotinoid classes, i.e., nitro- and cyano-substituted, in *F. candida*. To this end, we screened various genes to verify whether collectively their expression adhered to three criteria: (1) indicate exposure of both nitro- and cyano-substituted neonicotinoids, (2) in a concentration-dependent manner relate with the adverse effects of neonicotinoid exposure on *F. candida* fecundity, and (3) be reliable under synergistic interaction by CYP metabolic inhibition.

### PBO can be applied as a stress-test for both nitro- and cyano-substituted neonicotinoids

In this study, we applied PBO to determine the reliability of biomarkers in indicating the two major classes of neonicotinoids, i.e., nitro- and cyano-substituted, and as a model for synergistic interaction. In other words, we proposed PBO as a “stress-test” for biomarker robustness. The application of PBO in this manner was mainly based on earlier findings in different bee species (Beadle et al., 2019; Iwasa et al., 2004; Manjon et al., 2018). However, the genome of the honey bee has less redundancy in xenobiotic detoxification enzymes compared to other species (Claudianos et al., 2006), while *F. candida* has a genome with a diverse range of xenobiotic detoxification enzymes (Faddeeva-Vakhrusheva et al., 2017). Therefore, we first had to confirm that CYP-mediated metabolism had a comparative influence on neonicotinoid detoxification as in other species and also mediated differential toxicity of nitro- and cyano-substituted neonicotinoids. Our results show that PBO enhances the potency of both nitro- and cyano-substituted neonicotinoids and that this potency-enhancing effect is larger for the cyano-substituted thiacloprid. Our results are, therefore, in line with earlier findings in bees (Beadle et al., 2019; Gomez-Eyles et al., 2009; Manjon et al., 2018) and indicate that CYP detoxification mediates neonicotinoid similarly in *F. candida* compared to previously studied species

Moreover, we observed that PBO affects neonicotinoid toxicity at concentrations lower than the EC_1_ for PBO effects on springtail fecundity, i.e., 288 mg PBO kg^−1^ dry soil. Because PBO enhanced the potency of the neonicotinoids to springtail reproduction far below concentrations at which it becomes toxic itself, we may attribute the potency-enhancing effect of PBO on neonicotinoid toxicity to *F. candida* fecundity to the metabolic inhibition of CYP enzymes by PBO.

Because of these two findings, PBO can serve as a “stress-test” to determine if biomarkers remain reliable indicators of the exposure to two major classes of neonicotinoids even under synergistic interaction by CYP-inhibiting pollutants.

### Stability of biomarkers for neonicotinoid exposure

In our study, the three CYP genes did not adhere to any of the three biomarker criteria mentioned above, but mainly responded to the PBO treatment. Fent et al*.* (2020) surveyed the expression of two CYP genes in honey bee brains that were previously identified by Manjon et al*.* (2018) to metabolize imidacloprid and thiacloprid. However, these CYP genes were not differentially expressed at either low or high dosages of thiacloprid after 48 h exposure. Our results indicate that CYP genes associated with xenobiotic detoxification, i.e., *CYP6e2* and *CYP3A13*, were downregulated after exposure to thiacloprid and showed no significant response to imidacloprid. Based on our findings and those of Fent et al. (2020), it is doubtful that CYP genes involved in xenobiotic detoxification, even when involved in neonicotinoid detoxification in *F. candida* would respond to neonicotinoid exposure and could be used as biomarkers under our criteria. Therefore, we conclude that CYP genes are poor candidates to include in a panel of biomarkers for neonicotinoid exposure.

The genes *IPNS*, *VIT*, and *HSP70* in *F. candida* that have previously been shown to respond to variety of stress types (de Boer et al., 2009; Roelofs et al., 2012), and thereby could provide adherence of the biomarker panel to criteria 2, did not relate in a concentration-dependent manner to the adverse effect of neonicotinoids. Only two genes, when considered together, did adhere to all three criteria, *nAchR* and *SMCT*. Because PBO altered the expression of *nAchR* under co-exposure with imidacloprid and of *SMCT* under co-exposure with thiacloprid, we conclude that combined within a biomarker panel they provide a robust indication for cyano- or nitro-substituted neonicotinoid exposure, also under synergistic interaction of CYP inhibition (criteria 1 and 3).

These results confirm the potential of our approach to identify robust biomarkers for neonicotinoid toxicity, in the context of synergistic interactions with other pollutants. At the same time, the results also demonstrate that the majority of the prominent candidate-biomarkers proposed to date are not suitable. To supplement a biomarker panel that could include *SMCT* and *nAchR*, subsequent studies could aim at using high-throughput screening methods, such as transcriptomics, to identify additional biomarkers that relate concentration-dependently to higher levels of neonicotinoid exposure.

A thorough environmental risk assessment (ERA) of soils requires various lines of information on the physiochemical properties of the soil and the chemical presence, and on the ecological and toxicological impacts of soil pollution (Apitz et al., 2005). Providing support for these lines of evidence can be cumbersome and costly. In particular, in case of complex mixtures, chemical analysis of the soil can result in an underassessment of risk because it may not include all, biologically relevant, chemicals, and their degradation products (Escher et al., 2020). In addition, chemical analysis usually focuses on total chemical concentrations while risk is related to the biologically available fraction. Gene expression responses are immediate and specific to the type of pollution and can, thereby, provide accurate information on exposure, bioavailability, and bioaccumulation of contaminants in organisms even when no effects on phenotypic traits are observed (Lionetto et al., 2019). The ERA of pesticides in the soil is in particular pressing case, because most European agricultural soils are polluted with a mixture of pesticides and their derivates and physiochemical properties of soil can alter the bioavailability and, therefore, exposure of these pesticides’ mixtures (Pelosi et al., 2021; Silva et al., 2019; van Gestel, 2012). Gene expression biomarkers can help focusing the efforts of the risk assessors to the most offending samples and inform their further analyses, while providing biologically relevant information on the type, toxicity, and exposure of contaminants, single and in mixtures (Escher et al., 2020; Fontanetti et al., 2011; Lionetto et al., 2019; Shi et al., 2017).

## Conclusion

For the successful biomonitoring of a variety of neonicotinoids using gene expression, a panel of biomarkers have to be identified that remain robust indicators for the two main classes of neonicotinoids even under synergistic interaction by CYP inhibition. Our study demonstrated that PBO can be used to test the reliability of genetic expression patterns for both major classes of neonicotinoids. Subsequently, we used PBO as a tool to confirm the validity of *SMCT* and *nAchR* as indicators of neonicotinoid exposure even under synergistic interaction by CYP inhibition. The biomarkers can form the basis of rapid and cost-effective tools in biomonitoring of neonicotinoid exposure in soil.

## Supplementary information

ESM 1 (DOCX 210 kb)

## Data Availability

All data will be made available upon request to the authors. The authors are committed to publish material, such as R-code, and data alongside the publication.
