# Supplementary material for: Biomarker development for neonicotinoid exposure in soil under interaction with the synergist piperonyl butoxide in Folsomia candida
Source: Environ Sci Pollut Res Int. 2022 Jun 21;29(53):80897–909. doi: 10.1007/s11356-022-21362-z (PMC9596504; doi:10.1007/s11356-022-21362-z)
Supplement: Supplementary file 1 — (DOCX 210 kb) [file 11356_2022_21362_MOESM1_ESM.docx]

# Supplementary Information to:

**Biomarker development for neonicotinoid exposure in soil under interaction with the synergist piperonyl butoxide in *Folsomia candida***

Ruben Bakker^1^, Astrid Ekelmans^1^, Liyan Xie^1^, Riet Vooijs^1^, Dick Roelofs^1,2^, Jacintha Ellers^1^, Katja M. Hoedjes^1^, Cornelis A.M. van Gestel^1^

^1^ Amsterdam Institute for Life and Environment (A-LIFE), Faculty of Science, Vrije Universiteit Amsterdam, De Boelelaan 1085, 1081 HV Amsterdam, The Netherlands

^2^ Keygene N.V., Agro Business Park 90, Wageningen, 6708 PW, The Netherlands

#

Table S‑1: Summary of the primer sets used in quantitative PCR to determine the effects of neonicotinoid or piperonyl butoxide (PBO) exposure on the gene expression of Folsomia candida in LUFA 2.2 soil. Given are the name of the primer set used, the primer direction, oligo sequence, the description of the primer set target, the target gene id according to release version 50 (Cunningham et al., 2019) and the efficiency at which the sets amplify the DNA fragments at each cycle shown as percentages.

| **name** | **direction** | **oligo sequence** | **description** | **gene id** | **Efficiency (%)** |
| --- | --- | --- | --- | --- | --- |
| *YWHAZ* | Forward | CCTACAAAAACGTCGTCGGTG | *tyrosine 3-monooxygenase* | Fcan01_06830 | 89.3 |
|  | Reverse | TGTTGCTTTCGTTCGAACC |  |  |  |
| *ETIF* | Forward | TGATTCTGGAGATCTTCGCGAG | *eukaryotic transcription initiation factor 1A* | Fcan01_13627 | 94.9 |
|  | Reverse | ACAGTGCAAAGGATTTCCCGA |  |  |  |
| *CYP 3A13* | Forward | TTCCATGCAAGTCATCACATCAG | *Cytochrome P450 monooxidase 3A13* | Fcan01_20588 | 106.5 |
|  | Reverse | CGGAAACACAAAGATTCGTTCTG |  |  |  |
| *CYP 6a2* | Forward | GCGTTAAAAGCGAGGCAAGA | *Cytochrome P450 monooxidase 6a2* | Fcan01_00866 | 89.3 |
|  | Reverse | GCGATATCCACGTTCGAATTGT |  |  |  |
| *FE* | Forward | AGCTTTGGATCCCCTCCAATT | *methyl farnesoate epoxidase* | Fcan01_21605 | 92.9 |
|  | Reverse | CGGTTTTGGTCGTGGCTAAAT |  |  |  |
| *IPNS* | Forward | GACATGTCGGCAAAACTCCTTC | *isopenicillin N synthase* | Fcan01_27072 | 84.4 |
|  | Reverse | GGGTAGCGAATAAGTCGCACTG |  |  |  |
| *VIT* | Forward | CGTGAGACTTGAGTTCGTGCAC | *vitellogenin 1* | Fcan01_15308 | 100.3 |
|  | Reverse | GGACCATTCGTCTGTTGCAAAT |  |  |  |
| *HSP70* | Forward | TTGGTCGACGTAGCTCCACTCT | *Heat shock protein 70* | Fcan01_10020 | 98.1 |
|  | Reverse | TGGGCTTGTTTGCATGGAAT |  |  |  |
| *nAchR* | Forward | CGTGGACCAGGACAGAGAAA | *nicotinic acetylcholine receptor* | Fcan01_01431 | 85.6 |
|  | Reverse | TTGCAGACCCCCATAGTCTG |  |  |  |
| *SMCT* | Forward | ATGGTTTGGGTCGTTTCGTG | *sodium-coupled monocarboxylate transporter* | Fcan01_08638 | 90.6 |
|  | Reverse | CGGTTGTCCGTATTCGCTTG |  |  |  |

Table S‑2: Metrics for model selection of Generalized Additive Models (GAMs) between the null-model (only neonicotinoid smooth term) and full-model (neonicotinoid and piperonyl butoxide (PBO) smooth terms) used to analyze data on the effects of imidacloprid and thiacloprid on gene expression responses of Folsomia candida exposed in LUFA 2.2 soil in the absence or presence of different levels of PBO. Selected models are in bold under the “selected model”-column. The full model was accepted when the p-value of the F-test over the model fits was below or equal to 0.1. Abbreviations in the columns are as follows: R^2^, the coefficient of determination of the model; AIC, Akaike information criterion; gcv, general cross-validation; residual df, residual degrees of freedom; F, F-value; p, p-value. Names of the target genes listed in the target column are abbreviations for: cytochrome P450 monooxygenases (CYP) 3A13, CYP6e2, methyl farnesoate epoxidase (FE), Heat Shock Protein 70 (HSP70), isopenicillin N synthase (IPNS), vitellogenin-1 (VIT), nicotinic acetylcholine receptor-subunit alpha1 (nAchR), and sodium-coupled monocarboxylate transporter 1 (SMCT).

| **neonicotinoid** | **target** | **Selected  model** | **R^2^** | **AIC** | **deviation explained** | **gcv** | **residual df** | **F** | **p** |
| --- | --- | --- | --- | --- | --- | --- | --- | --- | --- |
| imidacloprid | *CYP6e2* | null | -0.02 | 188.50 | 0.01 | 91.21 |  |  |  |
|  |  | **full** | 0.59 | 154.70 | 0.62 | 81.77 | 2.00 | 28.64 | <0.01 |
|  | *CYP3A13* | null | -0.02 | 182.65 | 0.01 | 87.98 |  |  |  |
|  |  | **full** | 0.56 | 153.86 | 0.60 | 80.65 | 2.00 | 23.77 | <0.01 |
|  | *FE* | null | -0.03 | 162.65 | 0.00 | 78.68 |  |  |  |
|  |  | **full** | 0.73 | 116.61 | 0.75 | 64.19 | 3.23 | 30.76 | <0.01 |
|  | *IPNS* | **null** | 0.14 | 117.20 | 0.18 | 57.28 |  |  |  |
|  |  | full | 0.10 | 120.68 | 0.19 | 66.42 | 1.92 | 0.17 | 0.84 |
|  | *VIT* | null | -0.02 | 128.12 | 0.00 | 62.46 |  |  |  |
|  |  | **full** | 0.11 | 125.65 | 0.19 | 68.44 | 2.94 | 2.62 | 0.07 |
|  | *HSP70* | **null** | -0.03 | 101.87 | 0.00 | 50.11 |  |  |  |
|  |  | full | 0.08 | 100.61 | 0.17 | 57.76 | 3.12 | 2.20 | 0.10 |
|  | *nAchR* | **null** | 0.35 | 95.21 | 0.38 | 46.83 |  |  |  |
|  |  | full | 0.39 | 94.63 | 0.45 | 54.76 | 2.04 | 2.13 | 0.13 |
|  | *SMCT* | **null** | 0.65 | 83.38 | 0.67 | 41.94 |  |  |  |
|  |  | full | 0.66 | 84.87 | 0.70 | 50.87 | 2.95 | 1.20 | 0.33 |
| thiacloprid | *CYP6e2* | null | -0.03 | 181.19 | 0 | 87.28 |  |  |  |
|  |  | **full** | 0.53 | 155.75 | 0.57 | 81.32 | 2.67 | 15.76 | <0.01 |
|  | *CYP3A13* | null | -0.01 | 182.38 | 0.01 | 88.01 |  |  |  |
|  |  | **full** | 0.58 | 151.9 | 0.61 | 80 | 2 | 25.39 | <0.01 |
|  | *FE* | null | 0.05 | 149.28 | 0.1 | 72.31 |  |  |  |
|  |  | **full** | 0.6 | 119.53 | 0.64 | 65.88 | 2.29 | 21.33 | <0.01 |
|  | *IPNS* | **null** | 0 | 117.31 | 0.03 | 57.12 |  |  |  |
|  |  | full | -0.06 | 121.17 | 0.03 | 66.07 | 2 | 0.06 | 0.94 |
|  | *VIT* | null | 0.04 | 115.29 | 0.07 | 56.27 |  |  |  |
|  |  | **full** | 0.18 | 112.1 | 0.26 | 62.14 | 2.89 | 2.9 | 0.05 |
|  | *HSP70* | null | -0.03 | 90.59 | 0 | 44.5 |  |  |  |
|  |  | **full** | 0.31 | 78.02 | 0.37 | 46.89 | 2 | 9.36 | <0.01 |
|  | *nAchR* | **null** | 0.61 | 68.71 | 0.64 | 35.37 |  |  |  |
|  |  | full | 0.61 | 70.83 | 0.66 | 44.93 | 2 | 0.81 | 0.45 |
|  | *SMCT* | null | 0.68 | 81.73 | 0.7 | 41.49 |  |  |  |
|  |  | **full** | 0.71 | 80.11 | 0.75 | 49.19 | 2.01 | 2.57 | 0.09 |

Table S‑3: Nominal and measured concentrations of imidacloprid (IMI), thiacloprid (THIA) and piperonyl butoxide (PBO) in LUFA 2.2 soil, on the day of soil spiking and the end of the exposure, days 0 and 21, respectively. Recovery was calculated as the ratio of measured and nominal concentration and expressed as a percentage. The recovery was not calculated for the samples measured after 21 days exposure, shown as NA (not applicable).

|  | | neonicotinoid | | | | PBO | | | |
| --- | --- | --- | --- | --- | --- | --- | --- | --- | --- |
|  | sampling day | | Nominal  (mg kg^-1^ dry soil) | Measured  (mg kg^-1^ dry soil) | Recovery (%) | Nominal  (mg kg^-1^ dry soil) | Measured  (mg kg^-1^ dry soil) | Recovery  (%) |  |
|  |  | |  |  |  |  |  |  |  |
| IMI | 0 | | 0 | 0 | 0 | 0 | 0 | 0 |  |
|  | 0 | | 0.4 | 0.61 | 153 | 0 | 0 | 0 |  |
|  | 0 | | 0.4 | 0.62 | 156 | 1 | 0.66 | 66 |  |
|  | 0 | | 0.4 | 0.50 | 126 | 10 | 8.4 | 84 |  |
|  | 21 | | 0.4 | 0.53 | NA | 10 | 4.56 | NA |  |
|  |  | |  |  |  |  |  |  |  |
| THIA | 0 | | 0 | 0 | 0 | 0 | 0 | 0 |  |
|  | 0 | | 1 | 1.03 | 103 | 0 | 0 | 0 |  |
|  | 0 | | 1 | 0.9 | 90 | 1 | 1.19 | 119 |  |
|  | 0 | | 1 | 1.03 | 103 | 10 | 10.6 | 106 |  |
|  | 21 | | 1 | 0.32 | NA | 10 | 6.24 | NA |  |
|  |  | |  |  |  |  |  |  |  |

Table S‑4: Reference group (control) performance of *Folsomia candida* in toxicity tests with neonicotinoids with piperonyl butoxide (PBO) in LUFA 2.2 soil. Reference groups were exposed to soils only treated with demineralized water, pretreated with acetone or pretreated with acetone and either 1 or 10 mg PBO kg^-1^ dry soil, abbreviated as water, acetone and PBO 1 or PBO 10, respectively. Also added are the validity criteria according to the OECD guideline 232 (OECD, 2016). Only the reference group of the 1 mg PBO kg^-1^ treatment did not adhere to these criteria and therefore is marked in bold.

| **Compounds** | **Control type** | **Mean adult mortality (%)** | **Mean juvenile count** | **Coefficient of variance (%)** |
| --- | --- | --- | --- | --- |
| Imidacloprid and PBO | Water | 9 | 1055 | 14 |
|  | Acetone | 17 | 1085 | 12 |
|  | PBO 1 | 8 | 1090 | 18 |
|  | PBO 10 | 12 | 1137 | 12 |
| Thiacloprid and PBO | Water | 6 | 565 | 20 |
|  | Acetone | 2 | 532 | 17 |
|  | PBO 1 | 14 | 411 | **34** |
|  | PBO 10 | 12 | 607 | 12 |
| PBO | Water | 6 | 877 | 24 |
|  | Acetone | 8 | 781 | 21 |
| OECD validity criteria |  | <20 | >100 | <30 |

Figure S-1: The effects of piperonyl butoxide (PBO) on the fecundity of Folsomia candida after 21 days exposure in LUFA 2.2 soil. The juvenile counts are shown as circles, the solid line shows the fit of a three-parameter logistic model. Effect concentration (EC) for the reduction in juvenile counts by 10 % and 50 %, EC_10_ and EC_50_, are shown as a dark red diamond and a red dot, respectively. Whiskers show the 95% confidence interval estimators as obtained using the delta method.

****Figure S-2: Quantile-quantile plots of the residuals compared to their theoretical normal distribution. The residuals are from the Generalized Additive Models (GAMs) fitted on log2-transformed normalized gene expression measured with quantitative PCR (qPCR) on Folsomia candida exposed in LUFA2.2 soil to imidacloprid (panel collection A) and thiacloprid (panel collection B) and piperonyl butoxide for 48 hours. Quantile-quantile plots per target gene, their names are above the panels. The names are abbreviations for: cytochrome P450 monooxygenases (CYP) 3A13, CYP6e2, methyl farnesoate epoxidase (FE), Heat Shock Protein 70 (HSP70), isopenicillin N synthase (IPNS), vitellogenin-1 (VIT), nicotinic acetylcholine receptor-subunit alpha1 (nAchR), and sodium-coupled monocarboxylate transporter 1 (SMCT). Residuals are shown as dots on the panels, perfect agreement between the residuals and the normal distribution is shown as solid black lines, 95 % confidence intervals are shown as grey bands.

Figure S-3: Histogram frequency plots of residuals from Generalized Additive Models (GAMs) fitted on log2-transformed normalized expression from quantitative PCR (qPCR) measurements on the gene expression of Folsomia candida exposed to imidacloprid (collection of panels A) and thiacloprid (collection of panels B) and piperonyl butoxide in LUFA2.2 soil for 48 hours. Models per target gene as indicated by the name above each panel, which are abbreviations for: cytochrome P450 monooxygenases (CYP) 3A13, CYP6e2, methyl farnesoate epoxidase (FE), Heat Shock Protein 70 (HSP70), isopenicillin N synthase (IPNS), vitellogenin-1 (VIT), nicotinic acetylcholine receptor-subunit alpha1 (nAchR), and sodium-coupled monocarboxylate transporter 1 (SMCT). Frequency of the residual occurrence indicated by height of each bar. Value of the residual shown below the axes labelled with “residuals” using ticks. Bars should center in height around zero and decrease in size equally to both sized when the residuals follow the normal distribution.

**References:**

- Cunningham, F., Achuthan, P., Akanni, W., Allen, J., Amode, M. R., Armean, I. M., Bennett, R., Bhai, J., Billis, K., Boddu, S., Cummins, C., Davidson, C., Dodiya, K. J., Gall, A., Girón, C. G., Gil, L., Grego, T., Haggerty, L., Haskell, E., … Flicek, P. (2019). Ensembl 2019. Nucleic Acids Research, 47(D1), D745–D751. https://doi.org/10.1093/nar/gky1113
- OECD. (2016). Collembolan reproduction test in soil. Guidelines for Testing Chemicals 232, Organization for Economic Cooperation and Development, Paris.
